# *In situ* growth of large-area and self-aligned graphene nanoribbon arrays on liquid metal

**DOI:** 10.1093/nsr/nwaa298

**Published:** 2020-12-16

**Authors:** Le Cai, Wanzhen He, Xudong Xue, Jianyao Huang, Ke Zhou, Xiahong Zhou, Zhiping Xu, Gui Yu

**Affiliations:** Beijing National Laboratory for Molecular Sciences, CAS Research/Education Centre for Excellence in Molecular Sciences, Institute of Chemistry, Chinese Academy of Sciences, Beijing 100190, China; School of Chemical Sciences, University of Chinese Academy of Sciences, Beijing 100049, China; Applied Mechanics Laboratory, Department of Engineering Mechanics and Centre for Nano and Micro Mechanics, Tsinghua University, Beijing 100084, China; Beijing National Laboratory for Molecular Sciences, CAS Research/Education Centre for Excellence in Molecular Sciences, Institute of Chemistry, Chinese Academy of Sciences, Beijing 100190, China; Beijing National Laboratory for Molecular Sciences, CAS Research/Education Centre for Excellence in Molecular Sciences, Institute of Chemistry, Chinese Academy of Sciences, Beijing 100190, China; Applied Mechanics Laboratory, Department of Engineering Mechanics and Centre for Nano and Micro Mechanics, Tsinghua University, Beijing 100084, China; Beijing National Laboratory for Molecular Sciences, CAS Research/Education Centre for Excellence in Molecular Sciences, Institute of Chemistry, Chinese Academy of Sciences, Beijing 100190, China; School of Chemical Sciences, University of Chinese Academy of Sciences, Beijing 100049, China; Applied Mechanics Laboratory, Department of Engineering Mechanics and Centre for Nano and Micro Mechanics, Tsinghua University, Beijing 100084, China; Beijing National Laboratory for Molecular Sciences, CAS Research/Education Centre for Excellence in Molecular Sciences, Institute of Chemistry, Chinese Academy of Sciences, Beijing 100190, China; School of Chemical Sciences, University of Chinese Academy of Sciences, Beijing 100049, China

**Keywords:** graphene nanoribbons, template-free chemical vapor deposition, comb-like etching, self-alignment, *in situ* growth

## Abstract

Intrinsic graphene features semi-metallic characteristics that limit its applications in electronic devices, whereas graphene nanoribbons (GNRs) are promising semiconductors because of their bandgap-opening feature. However, the controllable mass-fabrication of high-quality GNR arrays remains a major challenge. In particular, the *in situ* growth of GNR arrays through template-free chemical vapor deposition (CVD) has not been realized. Herein, we report a template-free CVD strategy to grow large-area, high-quality and self-aligned GNR arrays on liquid copper surface. The width of as-grown GNR could be optimized to sub-10 nm with aspect ratio up to 387, which is higher than those of reported CVD-GNRs. The study of the growth mechanism indicates that a unique comb-like etching-regulated growth process caused by a trace hydrogen flow guides the formation of the mass-produced self-aligned GNR arrays. Our approach is operationally simple and efficient, offering an assurance for the use of GNR arrays in integrated circuits.

## Teaser

A comb-like etched patterns triggered by the trace hydrogen flow was used to in-situ grow large-area and self-aligned graphene nanoribbon arrays on liquid copper.

## INTRODUCTION

Graphene is an inspiring multifunctional material for application in next-generation microchip electronics and energy-related devices as a result of its advantageous physical properties [1,2]. For example, its ultrafast carrier migration affords graphene enormous prospects in field-effect transistors [3,4], but it is difficult to obtain a high on/off current ratio because of the absence of bandgap. To realize an extensive and pragmatic use of graphene in semiconductors, bandgap engineering of graphene has attracted increasing attention [5–7]. Graphene nanoribbons (GNRs), which introduce a bandgap by confining charge carriers in the lateral dimension and feature excellent optoelectronic properties, show huge prospects for application in optoelectronic devices [8,9]. Therefore, a robust nanotechnology to mass-fabricate GNRs of high quality and uniform orientation is highly desired.

Presently, the method of GNR preparation is underdeveloped, with common strategies including the cutting of carbon materials (graphene films, carbon nanotubes or graphite) and *in situ* growth on a specific substrate. The etching methods involve an *ex situ* process in which the use of low-resolution etching tools (electron beam [10,11], plasma [12,13] and metal nanoparticles [14,15]) inevitably results in rough edges for the GNRs. Meanwhile, the use of a mask to pattern the etched channels increases the risk of surface contamination. By contrast, *in situ* growth is promising for the fabrication of GNRs with smooth edges and clean surface. During the *in situ* growth process, the special substrates are indispensable materials to catalyze the decomposition of carbon sources or to define the ribbon width. Examples of *in situ* growth methods include: (1) the coupling and cyclodehydrogenation of linear dihalo polycyclic precursors on a single-crystal metal surface under ultrahigh vacuum conditions [16–18]; (2) surface graphitization on a specific crystal facet of SiC [19,20]; and (3) template-assisted chemical vapor deposition (CVD) on Ni nanobars [21,22], boron nitride trenches [23] or copper twin crystals [24]. These methods are inspiring but have a narrow scope: they require concession steps to prepare molecular precursors or pre-functionalize substrates as templates. Although scattered GNRs with lengths of hundreds of nanometers can be directly deposited on Sb-doped Ge (001) surface by the CVD technique [25], achieving GNR arrays is difficult because of the random nucleation. Thus, the preparation of GNR arrays requires a complex process to introduce templates for orientation alignment [26]. Therefore, controlling the growth of GNR arrays is still a challenge. The sub-micrometer length, alignment problem, low yield and cumbersome preparation process have retarded the integration of GNR arrays into optoelectronic devices. In this study, we seek to expand the scope of the GNR preparation method to obtain large-area GNR arrays with uniform alignment, high aspect ratio and smooth edges.

Hydrogen plays a dual role (catalyst and etchant) in the formation of graphene during the CVD process [27–29], thus affecting the graphene morphology, size and structure. For example, Ding's group revealed the function of hydrogen pressure in the growth of bilayer graphene, which clarified how hydrogen regulates the growth of CVD graphene [30]. Regarding hydrogen as an etchant, the increased flow rate promotes conversion from fractal to hexagonal hole etching modes after the methane supply is terminated [31–33]. Our previous studies have revealed the physicochemical underpinnings of the etching effect of hydrogen in modulating graphene patterns [31,34,35]. In addition, liquid metals as catalytic substrates tend to obtain high-quality and self-aligned two-dimensional materials because of the great surface uniformity without grain boundaries [36–39]. The Fu group synthesized superordered graphene structures by self-alignment on a liquid Cu surface, providing a new strategy for the precise assembly of two-dimensional materials [40]. These different growth behaviors of graphene are ascribed to the changes in growth conditions, including the growth substrate and carrier gas atmosphere. On the basis of the broad-scope strategies, we envision that the etching and self-assembly effects involved in the graphene growth are beneficial to the preparation of GNR arrays via template-free CVD.

In this work, we design a comb-like etching-regulated growth process to *in situ* fabricate GNR arrays on a liquid Cu surface for the first time. The comb-like patterns with linear and aligned etched channels transform the conventional growth mode of the two-dimensional film into a quasi-one-dimensional growth mode for the selective synthesis of GNR arrays without the assistance of a template. Moreover, the distinctive design allows for precise control over the width, edge structure and orientation of GNR arrays. Our method is an efficient and simple route to prepare large-area and high-quality GNR arrays.

## RESULTS AND DISCUSSION

### Large-area and self-aligned monolayer GNR arrays

**Figure 1.**
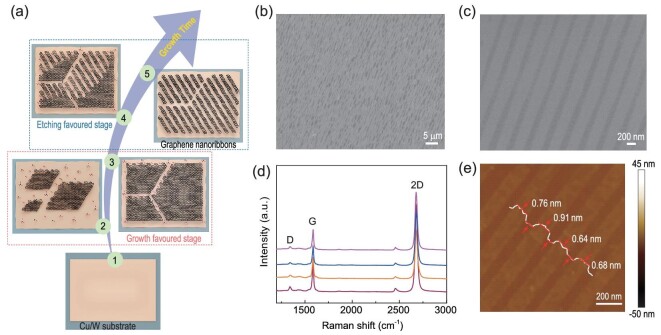
Large-area and self-aligned monolayer GNR arrays. (a) Schematic diagram for GNR array preparation via comb-like etching-regulated growth on liquid Cu surface. (b and c) SEM images of the large-area and self-aligned GNR arrays. (d) Typical Raman spectra of the monolayer GNR arrays transferred onto SiO_2_/Si substrate. (e) AFM image of the monolayer GNR arrays transferred onto SiO_2_/Si substrate. The growth conditions include a growth time of 10 min, growth temperature of 1120°C, and gas atmosphere of 0.8 sccm CH_4_, 6 sccm H_2_, and 930 sccm Ar.

The purposeful adjustment of hydrogen flow rate motivates a new discovery of comb-like etching-regulated growth process, and the formed linear and aligned etched patterns provide a rapid path to produce large-area, self-aligned and high-quality GNR arrays on a liquid Cu surface. A schematic of the preparation procedure is presented in Fig. 1a. After the growth process was completed, a large-area GNR array with uniform orientation was well dispersed over the resolidified Cu surface (Figs 1b and S1). The coverage of the GNR arrays was only limited by the Cu surface area, indicating the realization of mass production (density about 10^6^/cm^2^). The statistical size distribution of the GNR arrays showed a narrow window of dispersity, with widths and lengths in ranges of 100−200 nm and 2.5−3.5 μm, respectively (Fig. S2). The size deviation resulted from the existence of defects in the graphene film [35]. Moreover, the uniform orientation of GNR arrays benefited from the effect of self-assembly on the liquid Cu surface [41,42]. To validate the necessity for the liquid Cu catalyst, a polycrystalline solid Cu foil was used as the growth substrate for comparison. On solid Cu surface, graphene displayed cluttered etched patterns and no support for the formation of GNR arrays after sufficient growth time, as a result of disturbances of grain boundaries, steps and different crystal faces (Fig. S3a and b). From these results, we infer that liquid Cu as the substrate helps to form a comb-like etched pattern in graphene and orientational GNR arrays over the growth time, because of reduced interference from defects (Fig. S3c and d). Figure 1c shows a high-resolution scanning electron microscopy (SEM) image of the self-aligned GNR arrays, exhibiting both high quality and uniformity, with width of 110 ± 15 nm and smooth, sharp edges. In addition, the as-grown GNR arrays were transferred onto a SiO_2_/Si substrate to detect the thickness via Raman spectroscopy (Fig. S4). All of the stripes in the GNR arrays showed a typical single-layer feature: the intensity ratio of 2D to G peaks was in the range of 2−4, and the 2D peak was symmetric, with a full width at half-maximum of 31−33 cm^−1^ (Fig. 1d). A weak D peak existed, as the GNR width was far smaller than the diameter of the laser point (approximately 2 μm) [43]. Moreover, the AFM measurements confirmed that all of the GNRs were monolayer and featured a uniform orientation (height: < 1 nm, Fig. 1e). These data verify the reliability of our method for preparing large-area and self-aligned monolayer GNR arrays.

### Sub-10 nm GNR single crystal with high aspect ratio

**Figure 2.**
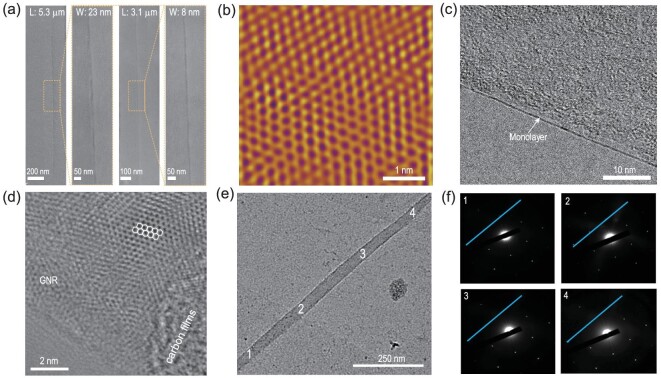
Narrow GNR single crystal with long length. (a) SEM images of the narrow GNRs with different widths obtained by prolonging the growth time (23 nm for 12 min; 8 nm for 14 min). L and W represent the GNR length and width, respectively. Yellow frames show the magnified images. (b) Filtered atomically resolved lateral force image of GNR showing the lattice structures. (c) TEM image of suspended monolayer GNR with a smooth and sharp edge. (d) High-resolution TEM image of GNR with zigzag edges. (e) TEM image of the single GNR transferred onto an amorphous carbon film surface. (f) SAED patterns obtained in different domains of GNR marked 1 to 4 in (e).

In addition to the realization of mass production and precise control over thickness and alignment, the changes in experimental conditions allow for controlling the GNR width and edge structure. A sub-10 nm GNR can open a bandgap up to 0.207 eV, and the bandgap is influenced by the GNR edge structures, width and length [10,44]. However, it is difficult to prepare sub-10 nm GNRs of length >1 μm following the *in situ* growth approaches reported in the literature. These short GNRs present problems for patterning electrodes during device fabrication. Fortunately, in this study, we achieved controllable preparation of narrow GNRs with a high aspect ratio. The width of the as-grown GNR decreased with increasing growth time: the width was cut down to approximately 23 nm with a length of 5.3 μm (aspect ratio of 230) after the GNR was grown for 12 min; the width further decreased to 8 nm with a length of up to 3.1 μm (aspect ratio of 387) after the GNR was grown for 14 min (Fig. 2a). The aspect ratios and lengths of the as-grown GNRs are higher than those of the CVD-synthesized GNRs in previous reports (with the highest aspect ratio of 70 and the longest length of 388 nm, Table S1). Moreover, the as-grown GNRs were of high quality: Figure 2b shows a filtered atomically resolved lateral force image, illustrating perfect, defect-free lattice structures. With regard to the edge type of GNR, previous studies suggest that CVD-grown graphene was etched by hydrogen to form zigzag edges with an angle of 120° [45]. In the current study, the comb-like etching behavior engendered similar angles of 120° at the terminal. Furthermore, theoretical calculations show that removing carbon dimers from the armchair sites is the least energy-costly etching process (Supplementary Note 1 and Fig. S5). A straight channel with zigzag sides could be formed after the etching process because the deflection of etched channels from armchair to zigzag sites needs more energy consumption. Besides, as the chemical driving force of etching increases (at high hydrogen flow), more removal processes can be activated, resulting in turns of the channels and a richer spectrum of etched patterns. Moreover, the narrow GNRs were transferred onto an amorphous ultrathin carbon film-loaded copper grid to detect the edge structures and crystal quality by transmission electron microscopy (TEM, Fig. S4). A suspended monolayer GNR with smooth and sharp edges was observed when the GNR was occasionally supported on a slightly damaged carbon film surface (Fig. 2c). Furthermore, the GNR lattice structures are shown in a high-resolution TEM image (Fig. 2d). Excluding the influence of drift distortion, the edge structure was identified as a zigzag motif, according to the arrangement of internal carbon atoms in the GNR. To determine the crystal quality of the GNR, the selected-area electron diffraction (SAED) patterns for the different GNR regions in Fig. 2e were obtained. The patterns show the same six-fold symmetric diffraction points without rotation angles (Fig. 2f). These results demonstrate that the as-grown GNR possessed a single-crystal nature without rotational boundaries. More importantly, each of the GNRs in the arrays also presented consistent diffraction spots without rotation angles, further confirming the self-assembly behavior of the GNR arrays grown on the liquid Cu surface and the uniform lattice structures (Fig. S6).

### Growth mechanism of GNR arrays

**Figure 3.**
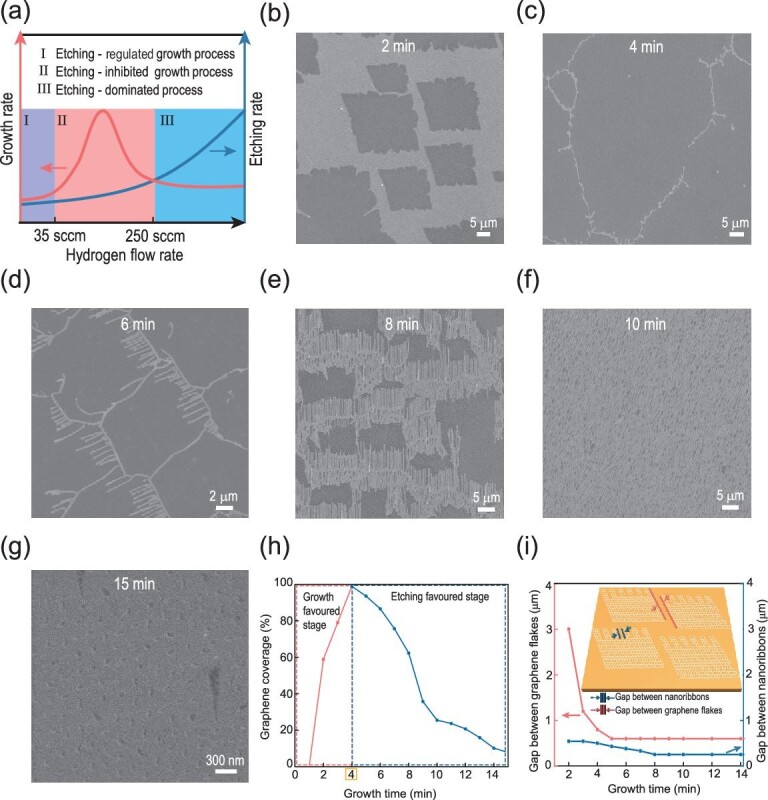
Growth behavior of graphene as a function of the hydrogen flow rate or the growth time. (a) Schematic diagram of the growth/etching rates for graphene as a function of hydrogen flow rate. The methane flow rate is 0.8 sccm. (b–g) SEM images of graphene after different growing times of (b) 2, (c) 4, (d) 6, (e) 8, (f) 10, and (g) 15 min. (h) Profile of graphene coverage as a function of growth time. (i) Profiles of gap between two neighboring graphene flakes and gap between two contiguous GNRs as a function of growth time, showing the dynamic equilibrium between growth and etching effects. The insets are schematic diagrams explaining these gaps. The growth conditions of all samples include a growth temperature of 1120°C and a gas atmosphere of 0.8 sccm CH_4_, 6 sccm H_2_, and 930 sccm Ar with different growth times.

To reveal the growth mechanism of self-aligned GNR arrays on the liquid Cu surface, we performed control experiments during the CVD process. As expected, the formation of GNR arrays was dominated by the hydrogen flow rate and growth time, and the influences of the Ar flow rate and the annealing process were negligible (Figs S7 and S8). Detailed experimental results on the influence of the hydrogen flow rate are shown in Fig. S9, and all of the growth conditions are summarized in Table S2. The changes in the hydrogen flow rate resulted in three growth behaviors for graphene when methane was set at a constant flow rate of 0.8 standard cubic centimeters per minute (sccm). At a very high hydrogen flow rate (>250 sccm), an etching-dominated process that failed to yield graphene was observed, suggesting that the etching rate overwhelmed the growth rate (Fig. S9a). At a moderate hydrogen flow rate (36–250 sccm), there were graphene films or hexagonal graphene flakes without observable etched regions, because of the lower rate of etching than growth, indicating an etching-inhibited growth process (Fig. S9b and c). This phenomenon is consistent with the findings of previous studies, in which no spontaneous, anisotropic etching occurred on graphene under these conditions [38–40]. At a low hydrogen flow rate (<35 sccm), the graphene morphology was affected by etching patterns, indicating an etching-regulated growth process and dynamic equilibrium between the etching and growth effects (Fig. S9d and e). Evidently, the graphene growth behavior was sensitive to the hydrogen flow rate: an increase in the flow rate elevated the growth and etching rate with different amplitudes (Fig. 3a). In addition, the further decrease in hydrogen flow rate facilitated the transformation of etching patterns from fractal (10–35 sccm, Fig. S9d) to comb-like (<10 sccm, Fig. S9e). More specifically, the comb-like pattern displayed linear and self-aligned etched channels, which is different from the reported fractal or hole etching patterns. The fractal patterns are prone to deviate from the original direction by 60° and 120°, and the spontaneous formation of hole etching patterns was suppressed by the methane supply during the graphene growth [32,46]. The formation of linear etched channels could be related to the activation energy barrier (*E_a_*). An Arrhenius analysis of the kinetics predicts that the activation barrier for branching is

$$\begin{equation*}
{E_a} = - {k_B}T\ln (p \times {10^{ - 13}}),
\end{equation*}$$

where *k_B_* is Boltzmann's constant, *T* is growth temperature and *p* is the probability for branching [35]. According to the equation, the activation energy barrier is inversely proportional to the probability for branching. At a trace hydrogen flow rate (<10 sccm), the fluctuation of hydrogen diffusion is energetically unfavorable because of the finite etchant (low probability for branching). Increasing the hydrogen flow rate (10–35 sccm) produces more hydrogen active molecules that significantly increase the probability for branching. This result agrees with the document that the linear etched channels deviate from the original direction to form fractal patterns under higher hydrogen flow as a result of more intensive fluctuation in diffusion [34].

Further investigations showed that the comb-like etching pattern was essential to prepare GNR arrays, whereas the fractal etching mode was inclined to construct hexagonal nanographene arrays [35]. Figure 3b–f shows the evolution of graphene flakes into self-aligned GNR arrays as a function of growth time under the regulation of comb-like etching patterns. However, nanographene fragments were obtained under excessive growth time (about 15 min, Fig. 3g), which will be discussed later. All of the growth conditions are listed in Table S3. From the evolution of GNR arrays, we observed that the coverage of graphene domains increased and then decreased with progressing growth time (Fig. 3h). According to this phenomenon, the *in situ* formation of GNR arrays operated through stepwise mechanisms, involving an intermediate growth-favored state and a subsequent etching-favored stage. The conversion mainly depends on the catalytic activity of liquid copper passivated by as-grown graphene, a process recognized as the self-limiting growth mechanism [47]. In the growth-favored stage, the abundant exposed Cu surface was replete with catalytic sites for generation of active hydrocarbon radicals. Therefore, the growth rate dominated over the etching rate until graphene sheets almost covered the whole Cu surface (in a growth time of approximately 4 min, Fig. 3c). Simultaneously, the etching effect of hydrogen led to the formation of rough edges and cracks, which were initially etching sites, in the graphene film. In the etching-favored stage, the growth effect was weakened by the decline in the active hydrocarbon radicals, because the liquid Cu surface was covered with as-grown graphene [48]. Thus, the etching effect was more prominent than the growth effect, and the comb-like etching patterns moved towards the interior of the graphene sheets from the rough edges as the growth time extended, leading to the formation of self-aligned GNR arrays. In addition, statistical data showed that the gap between two neighboring graphene flakes decreased to a stable value rather than to zero over the growth time, indicating that the etching effect was involved in the graphene growth and prevented the formation of graphene films. Meanwhile, the relationship between the gap of two adjacent GNRs (etched channels) and growth time was similar to that between the gap of two adjacent graphene flakes and growth time (Fig. 3i), showing the dynamic equilibrium between the growth and etching effects. Further evidence supporting the equilibrium is that the GNR arrays could be prepared only under the continuous supply of methane during the CVD process. When the methane supply was stopped at the later stages of growth and the hydrogen supply continued, the etching effect preferentially occurred in the already etched areas rather than moving towards the graphene interior (Fig. S10). Therefore, the movement of the comb-like pattern with straight and aligned etched channels into the graphene interior for formation of self-aligned GNR arrays benefits from the use of tenuous hydrogen flow and continuous supply of methane.

### Theoretical calculation for the GNR arrays

**Figure 4.**
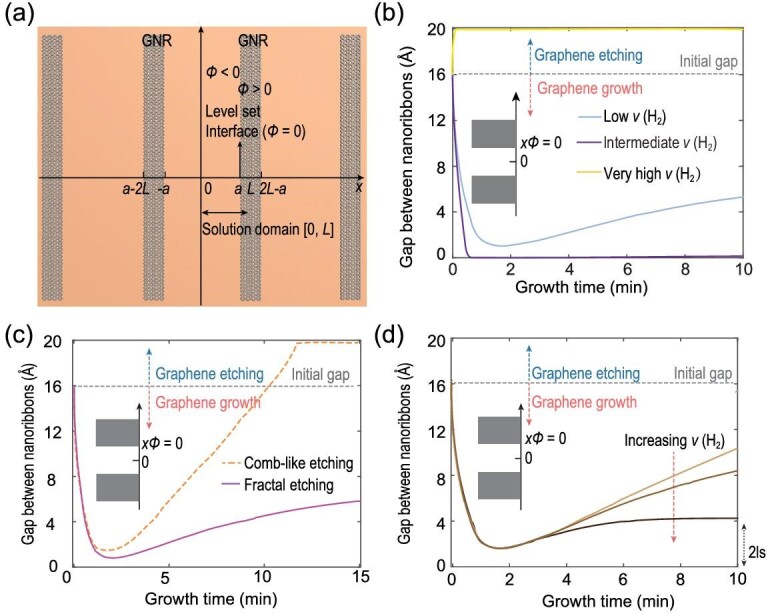
Theoretical model for depicting time evolution of graphene patterns under competitive growth and etching processes. (a) Schematic diagram of the 1D reaction-diffusion model under the assumption of uniformly spaced GNR arrays. (b) Time evolution of the gap distance under different hydrogen flow rates. Low *_v_*(H_2_), intermediate *_v_*(H_2_) and very high *_v_*(H_2_) correspond to experimental hydrogen flow rates of 10–35, 36–90, and >250 sccm. (c) Time evolution of gap distances for fractal (the parameter set ‘low’ in panel b) and comb-like (*r*_e_ increased by 2 times) etching-regulated processes. (d) Time evolution of gap distances under different hydrogen flow rates for the comb-like etching-regulated process. *F* and *r*_e_ values in the ‘low’ range correspond to experimental flow rates below 10 sccm.

To simulate the correlations between the graphene growth behavior and the hydrogen flow rate, we developed a 1D reaction-diffusion model, which corresponds to uniformly spaced GNR arrays. In this model, a field variable of free carbon concentration *c*(*x*, *t*) is defined in the domain of the solution spanned by the position *x* in the width direction and time *t*. With a mirror symmetry assumed, only the region [0, *L*] is considered in the analysis, where 2*L* is the period of GNR arrays, and the center of the gap regions between two contiguous GNRs is located at *x* = 0 (Fig. 4a). The adiabatic boundary conditions with zero mass flux are enforced at *x* = 0 and *L* following the mirror symmetry. A signed distance function (SDF, *φ* = *x −* *a*) is defined to identify graphene-covered regions (*φ* > 0), uncovered gap regions (*φ* < 0) and the interface (*φ* = 0). In experiments, the hydrogen flow rate directly controls the generation rate of free carbon atoms (*F*) from deposition, adsorption and decomposition processes on the exposed copper surface (*φ* < 0), as well as the etching rate (*r*_e_) from the as-grown graphene domain. It has been reported that the etching effect increases with hydrogen flow rate, while growth is promoted as hydrogen increases at low flow rates but decreases at high hydrogen flow rates [34,49]. Specifically, we assume an initial gap distance of 16 Å and 2*L* = 20 Å and choose the parameters *F*, *r*_e_ accordingly in our model (see details in Supplementary Note 1). This model reveals that the observed graphene morphology in the experiments was a result of the competitive growth and etching processes. The results in Fig. 4b align well with the experimental observations (Fig. S9), showing that hydrogen flow rate directly acts on the graphene growth behavior and decides the graphene final morphology.

In addition, we identified both comb-like and fractal etching modes in graphene at low hydrogen flow rates, an etching-regulated regime (Fig. 3b–g and Fig. S11). During the graphene growth, the comb-like etching patterns participated in the whole process, while fractal etching patterns were only activated in the later stage. This outcome can be explained by the fact that the growth effect was strengthened by the higher hydrogen flow rate, and the etching could proceed at edges and defects after the weakening of growth in the later stage. After an excessive growth time, the comb-like patterns tended to be almost fully etched for graphene, whereas the fractal patterns could remain stable, as equilibrium was achieved between etching and growth. The diffusion of hydrogen to the etching sites is larger and more scattered in the fractal mode, leading to depletion of hydrogen, which consequently limited the etching process (Fig. 4c). Additionally, in our model, the width of etched channels in the steady state with mass conservation in the domain of solution (2*l*_s_) decreased with the hydrogen flow rate in the comb-like etching mode (Fig. 4d), which is attributed to the dominance of the growth process in the etching-regulated region (Fig. 3a) and suggests a mechanism of width control for the GNR arrays.

### Width regulation of GNR arrays

**Figure 5.**
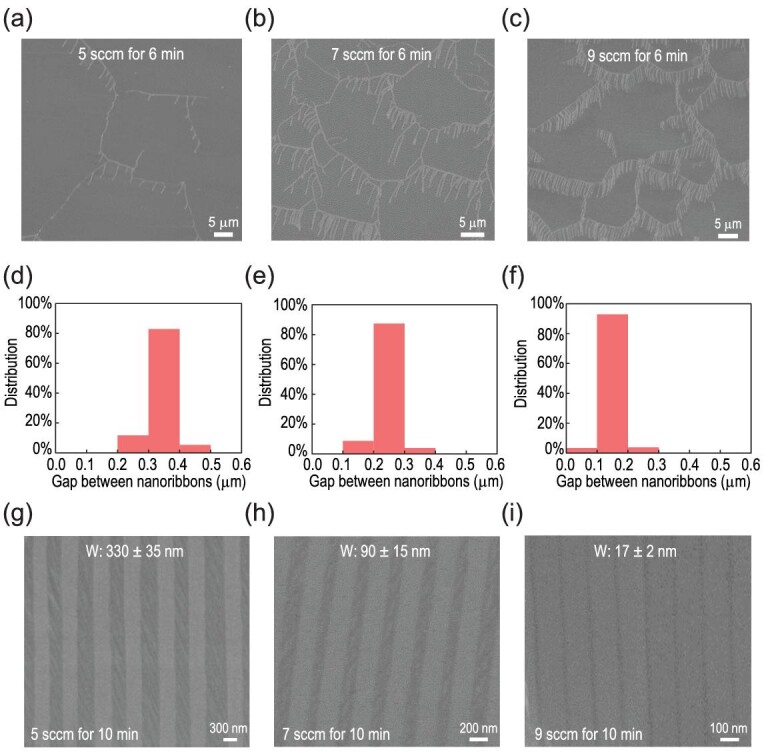
The width of GNR arrays regulated by the hydrogen flow rate. (a–c) SEM images of graphenes with different densities of etched channels, grown by variable hydrogen flow rates: (a) 5 sccm, (b) 7 sccm, and (c) 9 sccm; the other growth conditions include a growth temperature of 1120°C, growth time of 6 min and gas atmosphere of 0.8 sccm CH_4_ and 930 sccm Ar. (d–f) The steady-state width statistics of etched channels corresponding to the above comb-like etching patterns with different densities. (g–i) SEM images of GNR arrays with different widths obtained by prolonging the growing time (10 min) under the condition of the hydrogen flow rates in (a) to (c): (g) 5 sccm, (h) 7 sccm, and (i) 9 sccm.

As predicted by the calculation, with an optimized set of the hydrogen flow rates, we can control the density of the etched channels and, in turn, precisely modulate the width of the GNR arrays. Figure 5a–c shows an increase in the etched channels density with the increase in the hydrogen flow rate. This phenomenon is ascribed to the increased etched sites in graphene edges because a higher hydrogen flow rate will result in more hydrogen species in the active state. In addition, statistical data displayed that the steady-state widths of the etched channels were inversely proportional to the increase in hydrogen flow rate and were limited within a certain range (Fig. 5d–f), consistent with the simulation results. The narrower etched channels were caused by the growth of edges from the strengthened growth effect at higher hydrogen flow rates. Meanwhile, a minimized fluctuation ensures the uniformity of etched channels, which prompted us to grow GNR arrays with uniform width. By maintaining comb-like etching patterns with different densities for a growth time of 10 min, we achieved GNR arrays with different widths, which decreased with the increase in hydrogen flow (Fig. 5g–i). A combination of higher hydrogen flow rate and longer growth time resulted in successful preparation of the self-aligned GNR arrays, with a width of < 20 nm (Fig. 5i). This result reveals that the comb-like etching-regulated growth process plays a significant role in directly fabricating large-area, high-quality and self-aligned GNR arrays for further applications in electronic devices.

## CONCLUSION

In this study, we developed an *in situ* growth method to fabricate large-scale and self-aligned GNR arrays on liquid Cu surface without the assistance of a template. The unique designs for use of tenuous hydrogen flow and liquid Cu enabled the comb-like etching patterns to regulate the graphene growth, achieving a quasi-one-dimensional growth model for the GNR array formation. Through precise adjustment of the growth conditions, fine control over the width, edge structure and orientation of GNR arrays was realized. Moreover, the creation of sub-10 nm GNRs with a length of up to 3 μm opens an avenue for exploration of high-quality GNR arrays. The direct growth of GNR arrays streamlines the technological process and avoids the introduction of impurities and rough edges. Our method provides a reliable route for creating wafer-scale, high-quality and self-aligned GNR arrays, and thus has the potential to drive the development of graphene-based electronics.

## METHODS

### Materials synthesis

For this study, 100 μm-thick Cu foil (99.8% purity) was obtained from Sinopharm Chemical Reagent Co., Ltd., and 100 μm-thick W foil (99.95% purity) was purchased from Alfa Aesar. Before the graphene growth process, the Cu and W foils were successively cleaned by ultrasonication in deionized water, ethanol and acetone several times, and they were then immersed in dilute hydrochloric acid aqueous solution to remove oxide impurities. For the GNR growth, first, one piece of Cu foil (0.8 × 0.8 cm^2^) on W substrate (0.5 × 0.5 cm^2^) was placed in the center of a quartz tube. Afterwards, the whole system was rinsed by Ar gas at an ultrahigh flow rate (5000 standard cubic centimeters per minute, sccm) for about 30 s to remove the trapped air, followed by purging with a carrier gas of 200 sccm Ar and 10 sccm H_2_ for 10 min. Second, the furnace was heated up to a temperature of 1120°C within 1 h under the carrier gas flow (200 sccm Ar and 10 sccm H_2_). When the set temperature was reached, the substrates were annealed using pure Ar gas for a period to minimize the residual hydrogen induced in the heat-up stage. The GNR was grown at a temperature of 1120°C, with CH_4_ gas as the carbon source and a mixture of H_2_/Ar as the carrier gas. Finally, the CH_4_ flow was turned off, and the furnace was rapidly cooled to room temperature.

### Materials characterization

The as-grown GNR arrays were transferred onto a SiO_2_/Si substrate (SiO_2_ thickness: 300 nm) or an amorphous ultrathin carbon film-loaded copper grid via the electrochemical bubbling. The transfer process was performed as follows: First, the Cu/W substrate with GNR arrays was spin-coated with a 300 nm-thick poly(methyl methacrylate) (PMMA) film and then baked at 120°C for 2 h. Second, the GNR arrays/PMMA film was bubbled by hydrogen produced in the cathode. Finally, the PMMA was removed by acetone after being transferred onto the target substrate. The samples were characterized by scanning electron microscopy (SEM, Hitachi S-4800, 5 kV), optical microscopy (Olympus BX51), transmission electron microscopy (TEM, Jeol-2100F 200KV), Raman spectroscopy (HORIBA LabRAM HR Evolution, with laser excitation at 532 nm and a spot diameter of approximately 2 μm) and atomic force microscopy (AFM, Veeco Nanoman VS, operated in tapping mode).

## Supplementary Material

nwaa298_Supplemental_File
