# Supplementary material for: In situ growth of large-area and self-aligned graphene nanoribbon arrays on liquid metal
Source: Natl Sci Rev. 2020 Dec 16;8(12):nwaa298. doi: 10.1093/nsr/nwaa298 (PMC8692927; doi:10.1093/nsr/nwaa298)
Supplement: nwaa298_Supplemental_File [file nwaa298_supplemental_file.doc]

**Supplementary Materials for**

**In situ growth of large-area and self-aligned graphene nanoribbon arrays on liquid metal**

Le Cai^1,2^, Wanzhen He^3^, Xudong Xue^1^, Jianyao Huang^1^, Ke Zhou^3^, Xiahong Zhou^1,2^, Zhiping Xu^3^*, and Gui Yu^1,2^*

^1^Beijing National Laboratory for Molecular Sciences, CAS Research/Education Centre for Excellence in Molecular Sciences, Institute of Chemistry, Chinese Academy of Sciences, Beijing 100190, P. R. China;

^2^School of Chemical Sciences, University of Chinese Academy of Sciences, Beijing 100049, P. R. China;

^3^Applied Mechanics Laboratory, Department of Engineering Mechanics and Centre for Nano and Micro Mechanics, Tsinghua University, Beijing 100190, P. R. China

* **Corresponding authors**. E-mail: [yugui@iccas.ac.cn](mailto:yugui@iccas.ac.cn); [xuzp@tsinghua.edu.cn](mailto:xuzp@tsinghua.edu.cn)

*The Supplementary Materials Contain the Following Sections:*

**Supplementary Figures and Tables**

*Figures S1to S14*

*Tables S1 to S4*

**Supplementary Notes about Theoretical Calculation**

**References**

Supplementary Figures and Tables


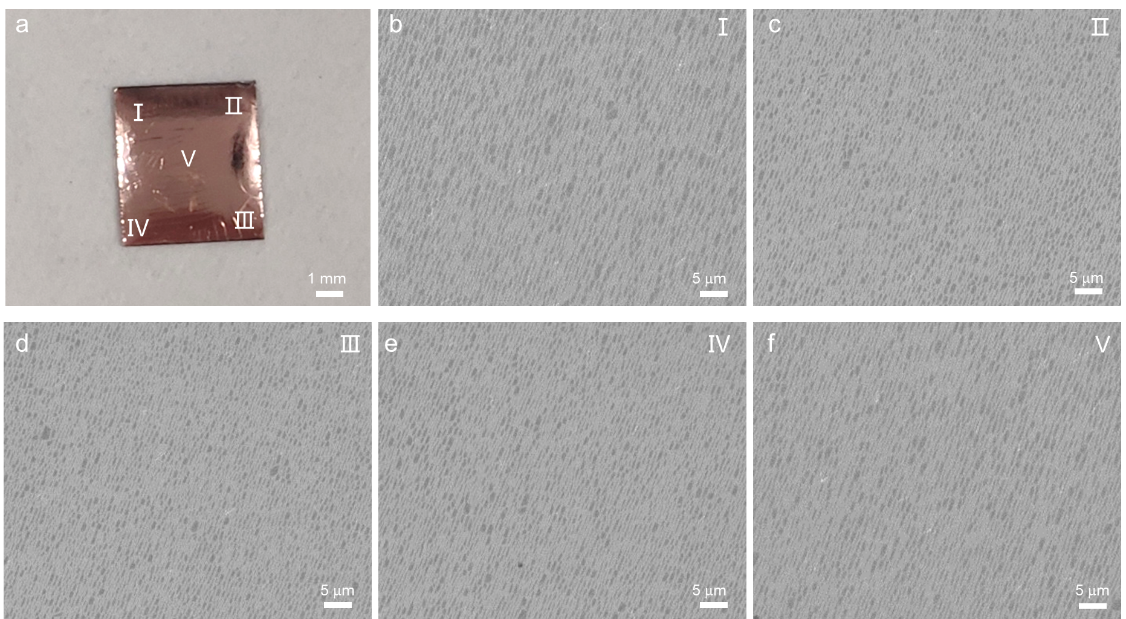


**Figure S1.**Large-area and self-aligned GNR arrays over the resolidified Cu surface prepared by the comb-like etching regulated growth process. (a) Photograph of 0.5 × 0.5 cm^2^ Cu/W sample with the GNR arrays after the in situ growth process. (b-f) SEM images of the self-aligned GNR arrays on different domains (I, II, III, IV and V) in the Cu/W sample of (a). The growth conditions include a growth time of 10 min, a growth temperature of 1120 °C, and a gas atmosphere of 0.8 sccm CH_4_, 6 sccm H_2_, and 930 sccm Ar.


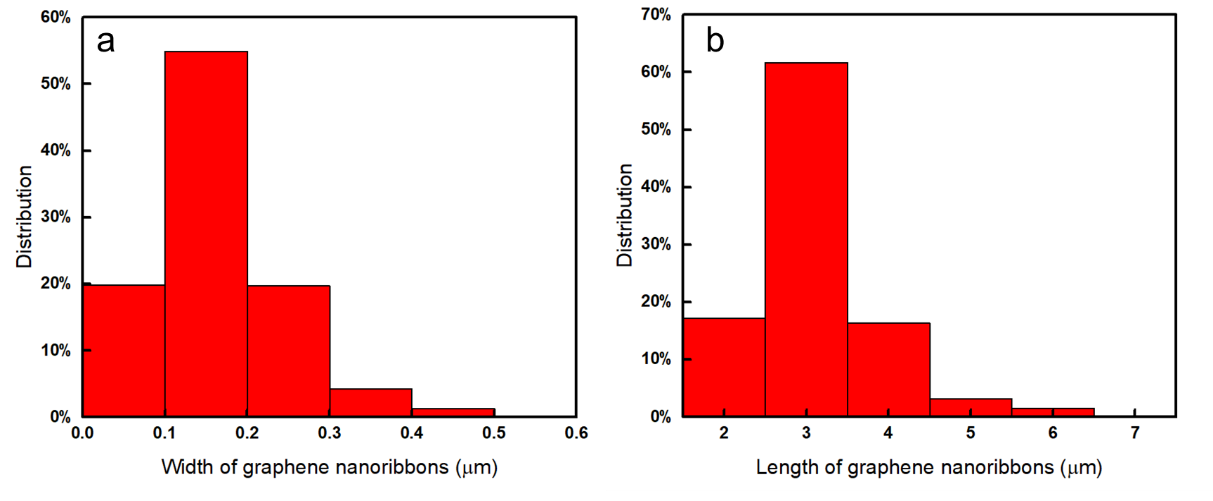


**Figure S2**. Size statistics of the large-area and self-aligned GNR arrays in Fig. S1. (a) The width statistics. (b) The length statistics.


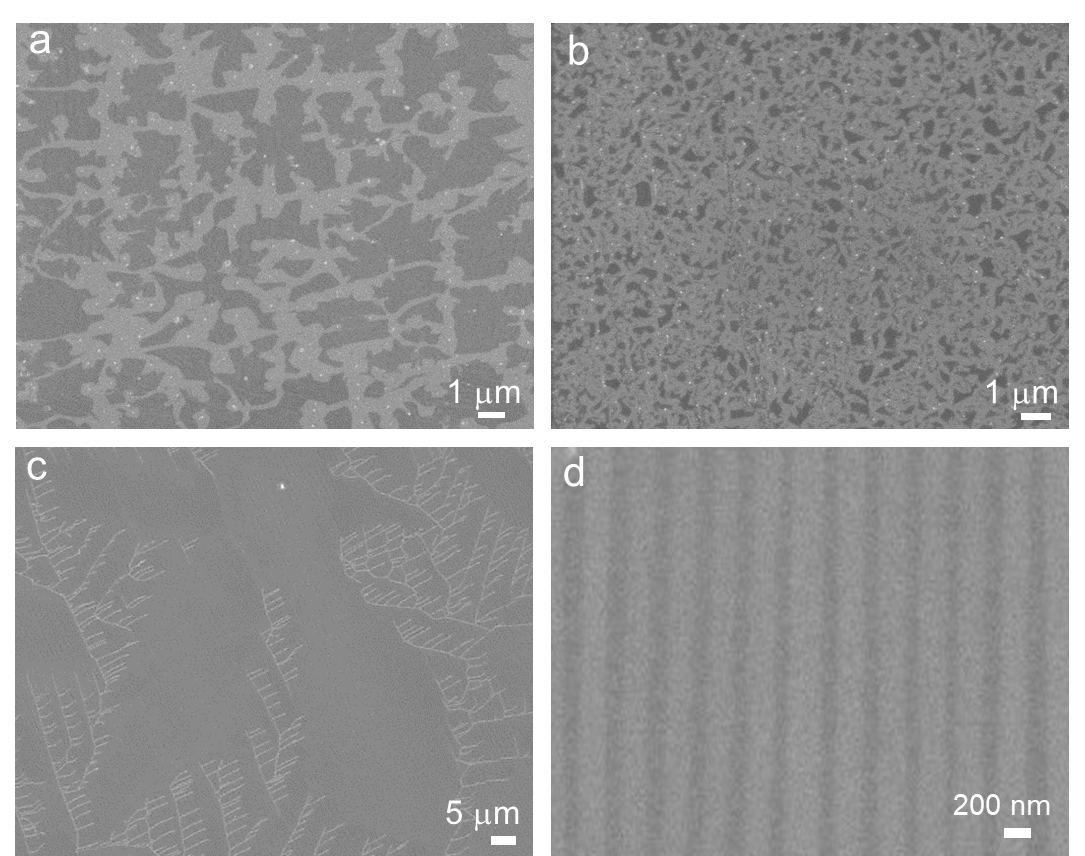


**Figure S3**. SEM images of the graphene grown on the solid and liquid copper surfaces. (a, b) Solid copper surface. The growth conditions were a growth temperature of 1035 °C, a gas atmosphere of 0.8 sccm CH_4_, 6 sccm H_2_, and 930 sccm Ar, and a growth time of 7 min for (a) and 10 min for (b). (c, d) Liquid copper surface. The growth temperature was 1120 °C, other conditions were consistent with the above with a growth time of 7 min for (c) and 10 min for (d).


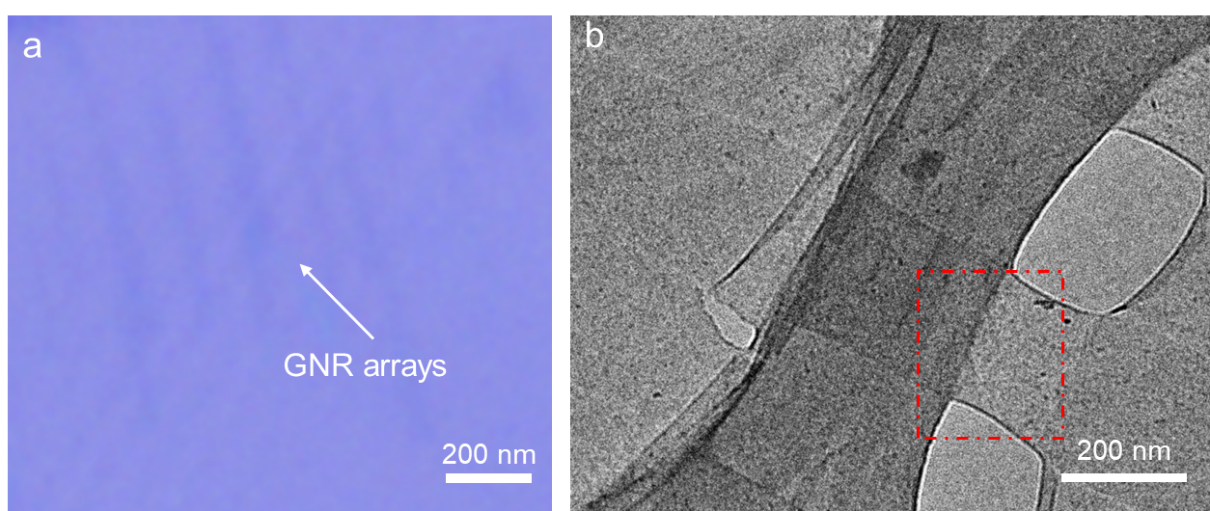


**Figure S4.** The transferred GNR arrays by electrochemical-assisted method. (a) Optical image of the self-aligned GNR arrays transferred onto the SiO_2_/Si substrate. (b) TEM image of the GNR arrays transferred onto the amorphous ultrathin carbon film-loaded copper grid. The red frame highlights the suspended GNR on the slightly damaged carbon film.

**Table S1.** Contrast between our method and other in situ growth ones reported in recent years about the quality of GNRs and catalytic substrate

| Substrate | Graphene nanoribbon | | | | Ref. |
| --- | --- | --- | --- | --- | --- |
|  | Layer number | Width (nm) | Length (nm) | Aspect ratio |  |
| Ni nanobar (width ranging from 20 to 100 nm) | N.A. | Sub-10 | 80-100 | 10 | 1 |
| Stepped SiC(0001) surfaces | Monolayer | 100-200 | N.A. | N.A. | 2 |
| Ni (100) deposited on MgO (100) single crystalline substrate | Monolayer | 20-50 | N.A. | ~ 20 | 3 |
| Hexagonal boron nitride with nano-trenches | Monolayer | Sub-10 | N.A. | N.A. | 4 |
| Cu twin crystals | Monolayer | ~100 | N.A. | N.A. | 5 |
| Au(111) substrate | Monolayer | 1.2 | 30-35 | 25 ± 4 | 6 |
| Ge(001) with graphene dots | N.A. | 40-80 | 286-388 | 17.5 ± 7.2 | 7 |
| Sb doped Ge(001) with miscut < 1° | Monolayer | Sub-10 | hundreds of nanometres | 70 | 8 |
| Liquid Cu | Monolayer | Sub-10 | ~3100 | ~387 | Our work |
| N.A. means no mention.  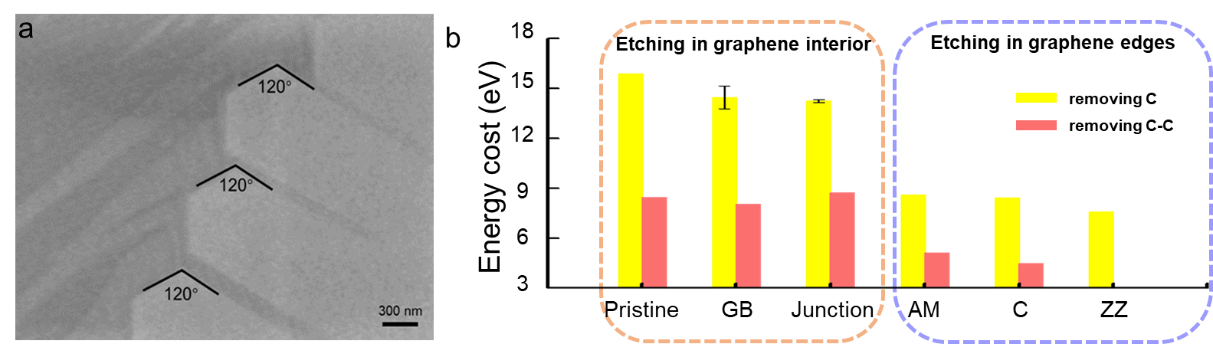  **Figure S5.** The edge type of the GNRs elaborated by experiment and theoretical calculations. (a) SEM image of partially completed GNR arrays with etching sharp turns of 120°. (b) The energy costs for removing a single carbon atom or a C–C dimer from graphene in different local environments, including the interior sites of a perfect single-crystal graphene (pristine), a grain boundary (GB), and a triple junction of three GBs (junction), as well as armchair (AM), chiral (C), and zigzag (ZZ) edges of graphene (Figures S12-13). | | | | | |


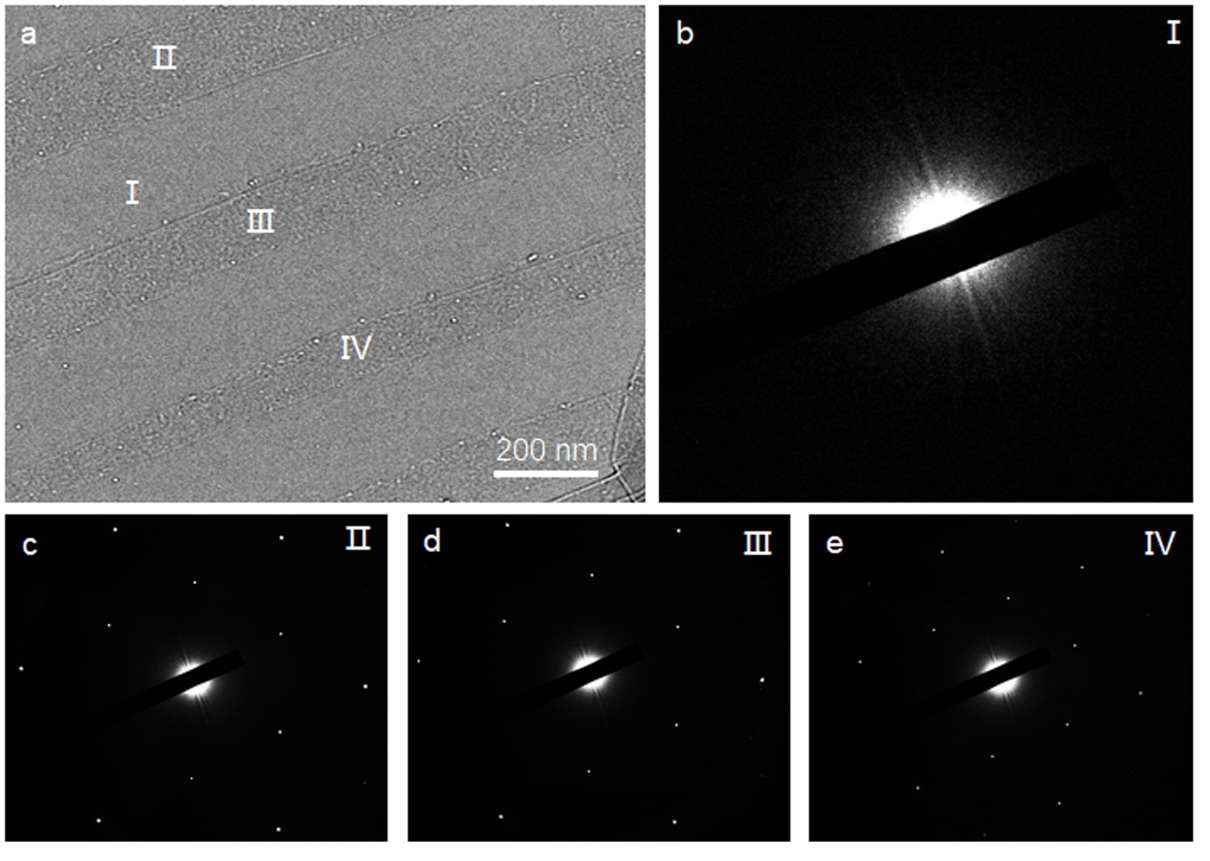


**Figure S6.** Self-aligned GNR arrays with single-crystal feature. (a) TEM image of the GNR arrays on an amorphous ultrathin carbon film-loaded grid. The light region (I) is a carbon film and the dark regions (II-IV) are GNRs. (b) SAED pattern of the ultrathin carbon film. The result suggests that amorphous carbon film does not affect the diffraction pattern of GNR. (c-e) SAED patterns of the different GNRs in the arrays marked as II-IV, respectively.


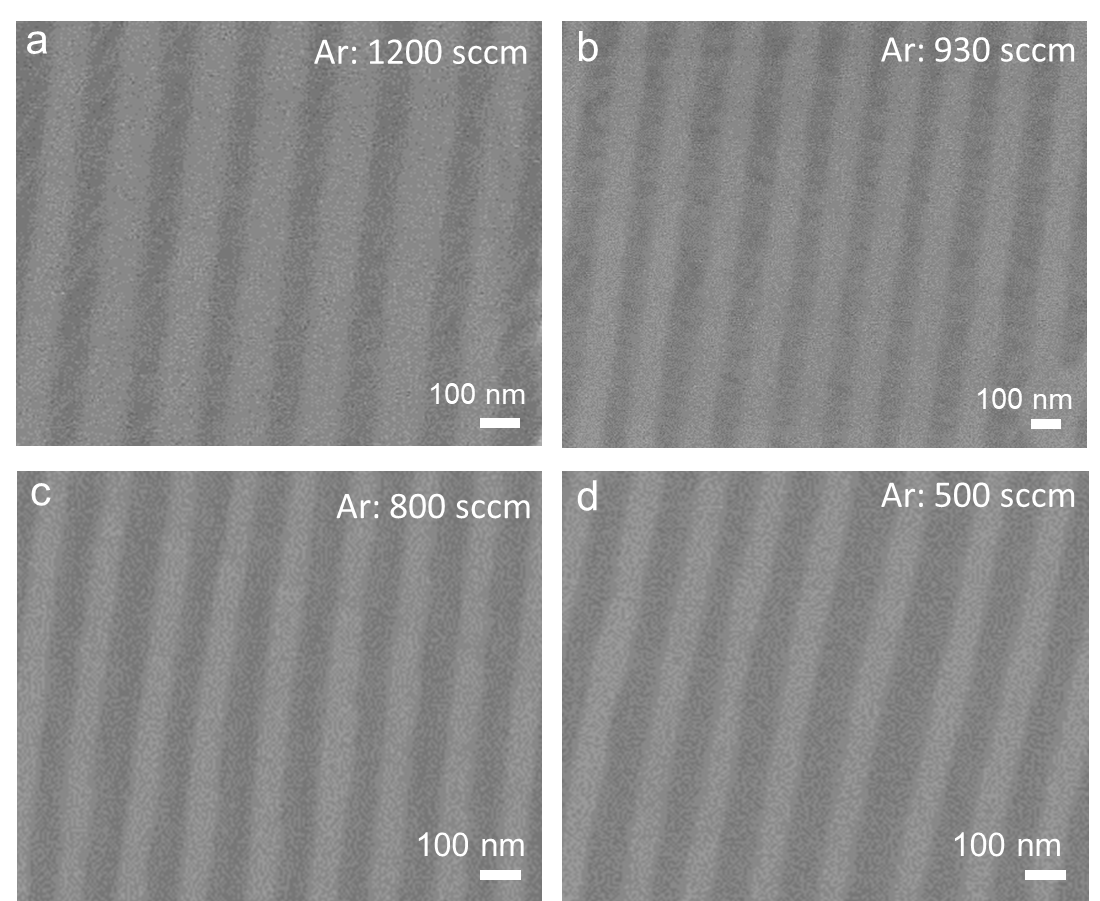


**Figure S7**. SEM images of the GNR arrays grown on a liquid Cu surface by CVD at different argon flow rates in the growth stage. (a) 1200 sccm. (b) 930 sccm. (c) 800 sccm. (d) 500 sccm. Other growth conditions are the same (0.8 sccm CH_4_ and 9 sccm H_2_ at 1120°C for 9 min).


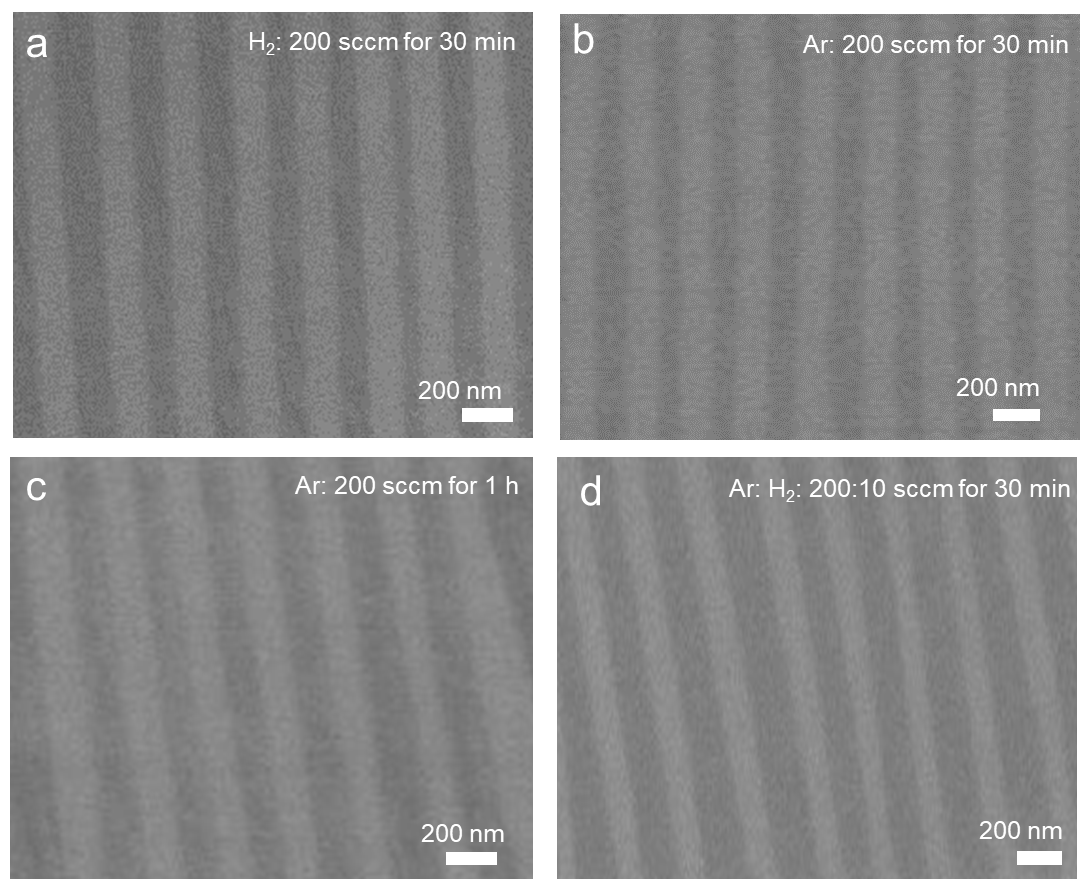


**Figure S8.** SEM images of the GNR arrays grown on the liquid Cu surface by CVD under different annealing conditions for the Cu/W substrate before graphene growth. (a) Hydrogen atmosphere (200 sccm) for 30 min. (b) Pure argon gas (200 sccm) for 30 min. (c) Pure argon (200 sccm) for 1 h. (d) Mixed gas of argon (200 sccm) and hydrogen (10 sccm) for 30 min. Growth conditions of GNRs are the same with a gas atmosphere of 0.8 sccm CH_4_, 8 sccm H_2_, and 930 sccm Ar, growth tempareture of 1120°C, and growth time of 9 min.


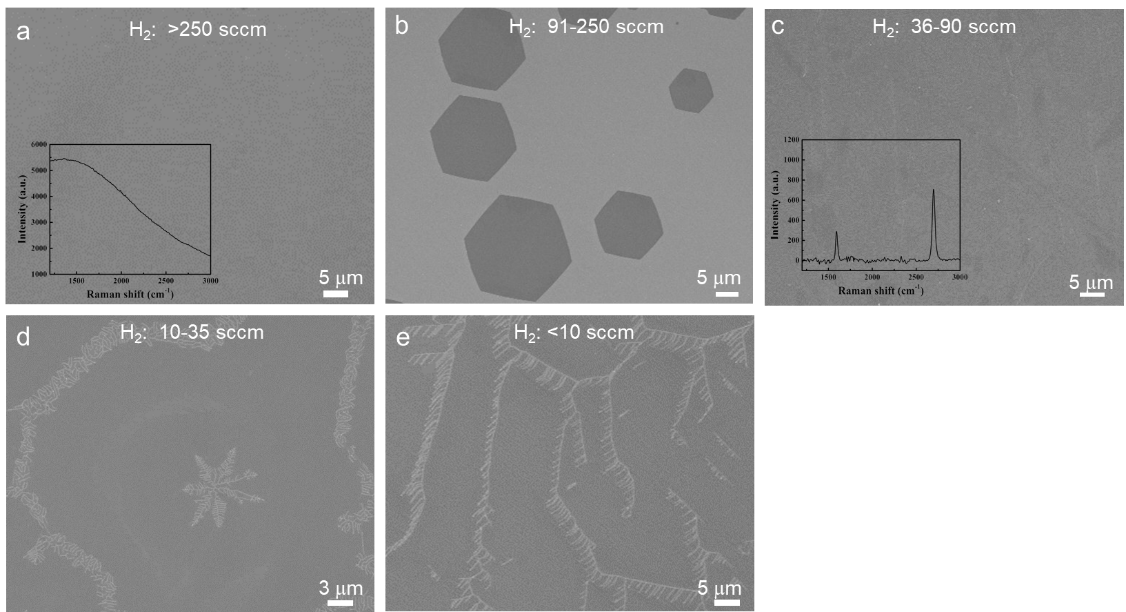


**Figure S9.** SEM images of the graphene grown by CVD at different hydrogen flow rates. (a) The etching-dominated process when hydrogen flow rate is higher than 250 sccm. Inset is the Raman spectrum to show the absence of graphene film on the Cu surface. (b, c) The etching-inhibited growth process at hydrogen flow rate of 35-250 sccm. (b) Hexagonal graphene flakes without etching patterns. (c) Monolayer graphene film without etching patterns. The inset is the Raman spectrum. (d) Fractal etching pattern and (e) comb-like etching pattern in the etching-regulated growth process at hydrogen flow rate of lower than 35 sccm.

**Table S2**. Detailed growth conditions of the samples for disclosing the relationship between the growth behaviour of graphene and the hydrogen flow rate

| **Growth temperature (°C)** | **CH_4_ flow rate (sccm）** | **Ar flow rate**  **(sccm)** | **H_2_ flow rate**  **(sccm)** | **Growth behaviour** | **Graphene (growing about 10 min)** |
| --- | --- | --- | --- | --- | --- |
| **1120** | 0.8 | 930 | <10 | Comb-like etching regulated growth process | GNR arrays |
|  |  |  | 10-35 | Fractal etching regulated growth process | Graphene fragments |
|  |  |  | 36-90 | Etching-inhibited growth process | Graphene film |
|  |  |  | 91-250 | Etching-inhibited growth process | Hexagonal graphene flakes |
|  |  |  | >250 | Etching-dominated process | No graphene |

**Table S3.** Parameters of graphene obtained under different growth conditions

| Growth temperature (°C) | Gaseous atmosphere  (sccm) | Growth time  (min) | Average gap between two adjacent graphene flakes (μm) | Average gap between two adjacent GNRs (μm) | Graphene coverage （%） |
| --- | --- | --- | --- | --- | --- |
| 1120 | CH_4_:H_2_:Ar  0.8:6: 930 | 2 | 3.0 | 0.54 | 62.83 |
|  |  | 3 | 1.2 | 0.54 | 79.04 |
|  |  | 4 | 0.80 | 0.50 | 98.91 |
|  |  | 5 | 0.60 | 0.40 | 98.15 |
|  |  | 6 | 0.60 | 0.40 | 97.65 |
|  |  | 7 | 0.60 | 0.35 | 95.71 |
|  |  | 8 | 0.60 | 0.25 | 66.34 |
|  |  | 9 | 0.60 | 0.25 | 35.75 |
|  |  | 10 | 0.60 | 0.25 | 25.55 |


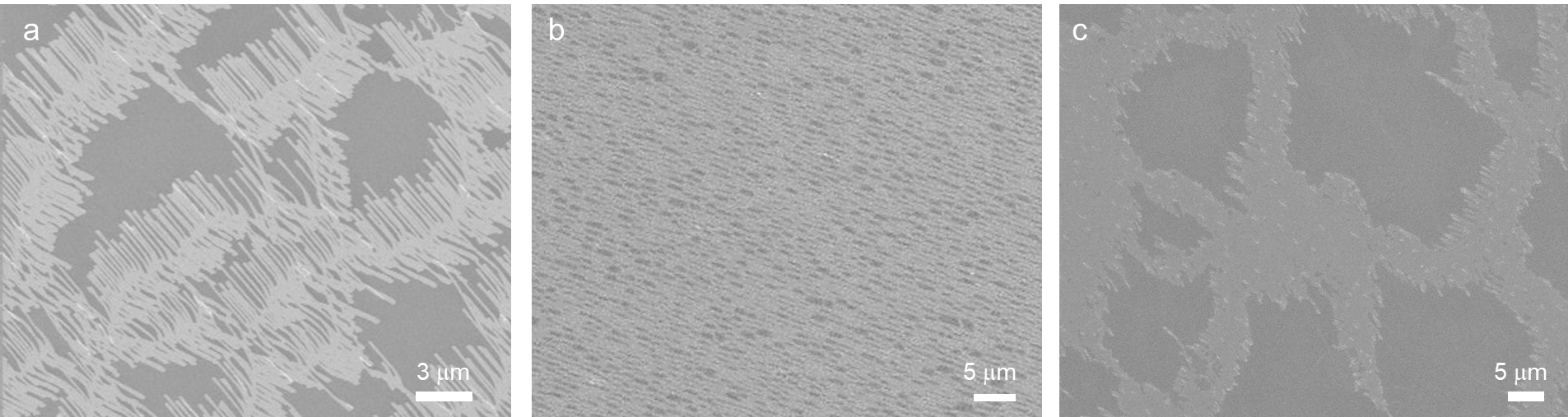


**Figure S10.** SEM images of the graphene grown with and without the continuous supply of methane in the whole CVD growth process. (a) Partly formed GNR arrays obtained by maintaining the continuous supply of CH_4_ for growing 8 min. (b) Completely formed GNR arrays obtained by maintaining the continuous supply of CH_4_ for growing 10 min. (c) Residual graphene fragments obtained by maintaining the supply of methane for 8 min and stopping its supply for etching 2 min. Other conditions are the same with a growth temperature of 1120 ^o^C and a gas atmosphere of 0.8 sccm CH_4_, 6 sccm H_2_, and 930 sccm.


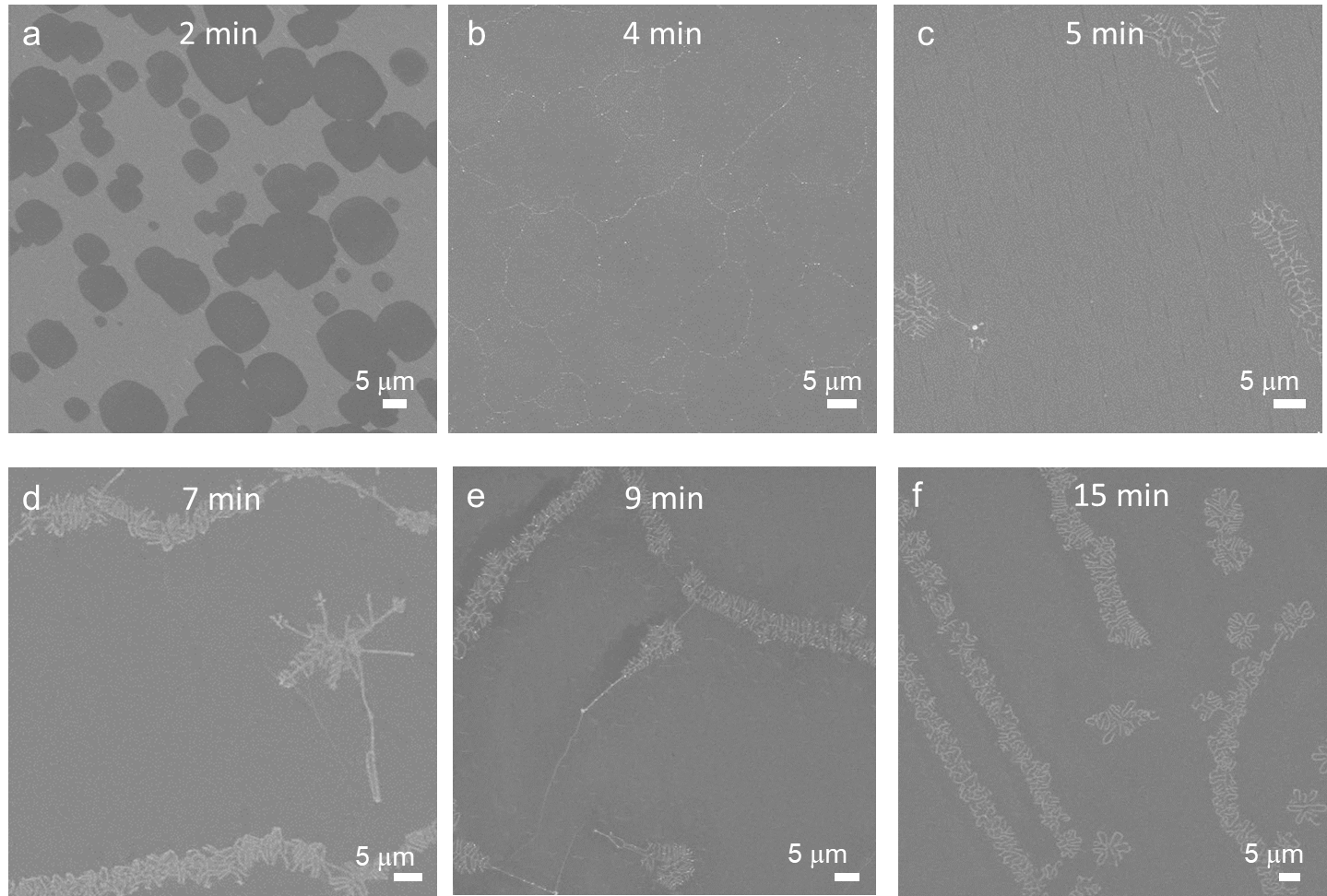


**Figure S11.** SEM images of the graphene prepared in fractal etching regulated growth process with different growth time. (a) 2 min. (b) 4 min. (c) 5 min. (d) 7 min. (e) 9 min. (f) 15 min. Other conditions are the same with a growth temperature of 1120 ^o^C and a gas atmosphere of 0.8 sccm CH_4_, 15 sccm H_2_, and 930 sccm.

**Supplementary Note 1 about Theoretical Analysis**

**1.1 Energy Costs of the Etching Processes**

***First-Principles Calculations.*** The Bronsted-Evans-Polanyi (BEP) rule suggests a positive correlation between the energy cost of an etching process and the activation energy.^9-10^ Consequently, to explore the kinetics of etching, we calculated the energy costs (Δ*E*’s*)* to remove a single carbon atom or a carbon dimer from different sites in graphene, which is defined as the energy difference between carbon atom and dimer in graphene in the gas phase.

We performed spin-polarized density functional theory (DFT) based first-principles energy calculations. The Perdew-Burke-Ernzerhof (PBE) parameterization of generalized gradient approximation (GGA) was used for the exchange-correlation functional.^11^ Projector augmented wave (PAW) potentials were used for the ion-electron interactions.^12^ All calculations were done by using the Vienna ab-initio simulation package (VASP) with an energy cut off of 500 eV for the plane-wave basis-sets.^13^ The criterion of total energy convergence for electronic self-consistency field (SCF) calculations is 10^−6^ eV. In geometry relaxation calculations, the force on atoms is converged below 0.02 eV/Å and the total energy is converged to within 10^−4^ eV. Periodic boundary conditions (PBCs) were applied in three directions, with a vacuum layer of 20 Å used for isolation in directions along which the structures are non-periodic. The *k*-space is sampled by a Monkhorst-Pack grid with a line density of ~ 45 points per Å.


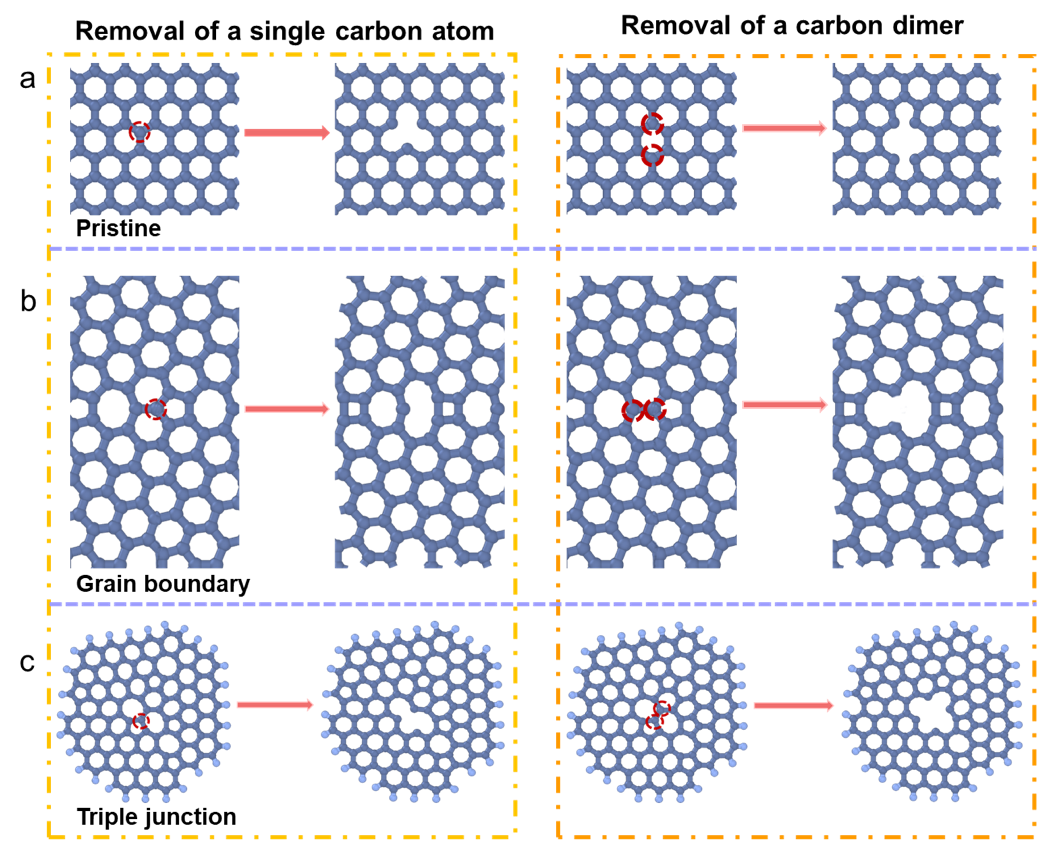


**Figure S12.** Models of removing a carbon atom and a carbon dimer from (a) a perfect single-crystal graphene interior, (b) a grain boundary (GB), and (c) a triple junction of three GBs. The red circles mark the atoms to be etched.

The energy cost of mono- or di-vacancies in graphene is defined as

Δ*E* = [*E*_etched_ + (*E*_monomer_ or *E*_dimer_)- *E*_g_]/*N*_C_ (S1)

where *E*_etched_ is the energy of etched graphene, *E*_monomer_ or *E*_dimer_ is the energy of removed carbon monomers (dimers) in the gas phase, *E*_g_ is the energy of graphene structures before etching, and *N*_C_ is the number of C atoms etched.

***Results and Discussion.*** We studied etching processes that remove monomers and dimers (*N*_C_ = 1, 2, see Table S4 and Figure S5b) only, and other etching processes are considered as composite steps of these two fundamental ones. For interior regions in graphene, we considered sites in the single-crystal graphene (pristine) region, the grain boundary (GB, *θ* = 21.8^o^, two choices to remove a carbon atom), and the 120^o^ triple junction of three GBs (junction, two choices to remove a carbon atom) are considered. For graphene edges, the armchair (AM), chiral with kinky angle of 10.9° (C), and zigzag (ZZ) edges are considered. Removing a dimer from ZZ edges leaves a carbon atom with only one bond, which is a high-cost process that is excluded in our discussion. The initial and etched structures after relaxation are shown in Figures S12-13. For periodic structures, the supercell is not relaxed by assuming that the stress effect can be ignored.

The results (Figure S5b) indicate that removing a carbon atom or carbon dimers from the edges is always less costly than those from the interior regions. The dominant etching process is to remove dimers from armchair sites at the armchair or chiral edges, followed by removing a single atom from the zigzag edges that leaves two kink armchair sites behind. Considering that the density of armchair sites at armchair edges is the highest, parallel etching processes could be activated, leaving the armchair edges etched at the highest rate. For the zigzag edges, removal of the first carbon atom will be the rate- limiting process, after which the armchair sites at the kinks could be etched step by step. A straight channel with zigzag edges could thus be formed after the etching process. Furthermore, as the chemical driving force of etching increases, more


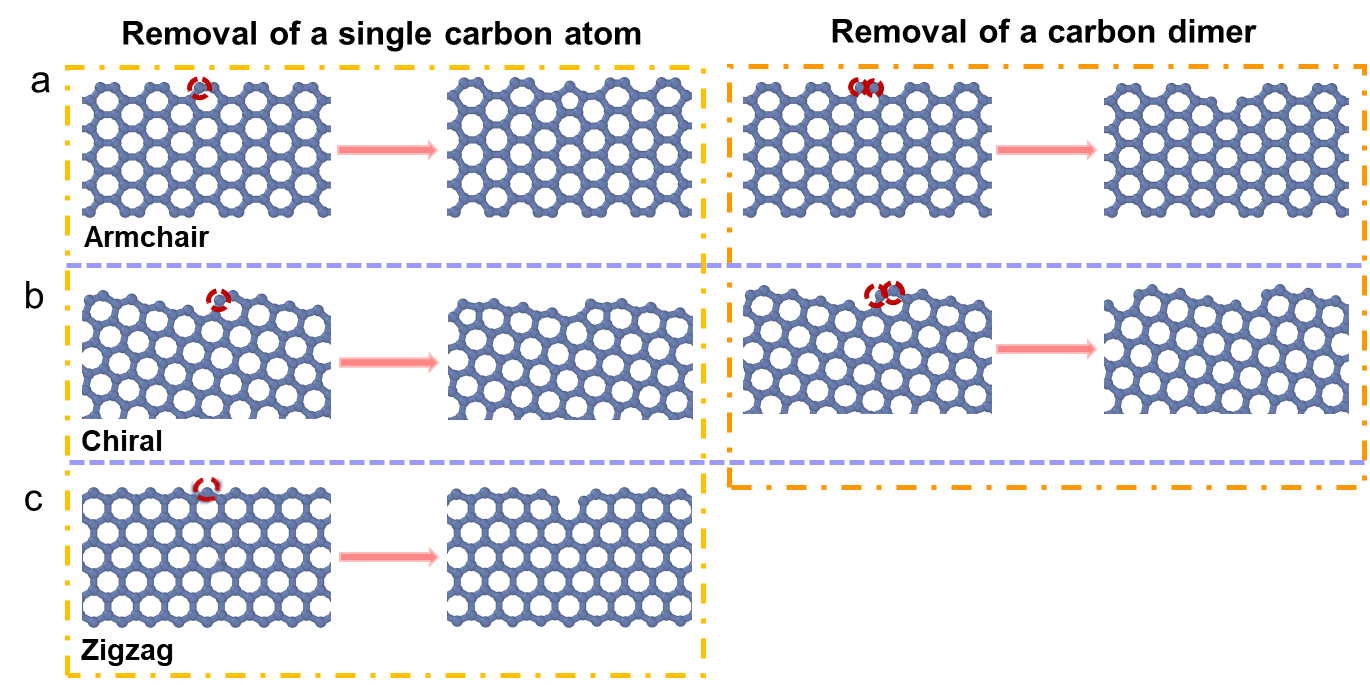


**Figure S13.** Edge structures of graphene before and after removing one and two carbon atoms from (a) armchair, (b) chiral, and (c) zigzag edges. The red circles mark the atoms to be etched.

removal processes can be activated, resulting in, for example, turns of the channels and a richer spectrum of etched patterns, which may explain the experimental findings for the fractal patterns.

**Table S4.** Energy costs for removing one C atom or a C-C dimer from graphene at different sites

| structures | Energy cost for removing one C atom (eV) | Energy cost for removing a C-C dimer (eV) |
| --- | --- | --- |
| Removal from interior graphene regions | | |
| pristine | 15.89 | 8.45 |
| GB | 14.46 ± 0.69 | 8.05 ± 0.00 |
| junction | 14.24 ± 0.08 | 8.72 |
| Removal from graphene edges | | |
| armchair (AM) | 8.60 | 5.11 |
| chiral (C) | 8.41 | 4.46 |
| zigzag (ZZ) | 7.58 | ­ |
|  | | |

**1.2 Graphene Growth/Etching Patterns**

***Models and Methods.*** We introduced a one-dimensional (1D) reaction-diffusion model for equally-spaced GNR arrays in the growth/etching processes, which is solved numerically by using the finite difference (FD) method where the interfaces are tracked through level sets.^14^ In the gap region (-*a* < *x* < *a*) between two neighbouring GNRs, the substrate is exposed for gas (e.g. CH_4_) deposition, adsorption, and decomposition, as well as evaporation and surface diffusion of free carbon, while in the region covered by graphene, the decomposition processes are inhibited owing to the lack of catalytic sites. A signed distance function (SDF, *ϕ = x - a*) was introduced to distinct the gap and covered regions. The adiabatic boundary conditions (BCs) with vanishing mass flux are enforced at both *x* = 0 and *x* = *L* by following the mirror symmetry with uniform widths of etched channels and GNR arrays (Figure 4a).

In the gap region, the free carbon concentration field is determined by the reaction and diffusion on the uncovered copper substrate. The governing equation is


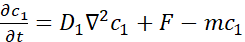
 (S2)

where *D*_1_ is the surface diffusivity of free carbon on the catalytic substrate, *F* is the deposition rate of free carbon atoms, and *m* is the evaporation rate of free carbon atoms. The three terms on the right side of **Eq. S2** are contributions from surface diffusion of free carbon, deposition, adsorption and decomposition from carbon sources, and evaporation of free carbon atoms. The adiabatic BC is at *x* = 0.

In the graphene-covered region where catalytic reactions are prohibited, only diffusion and evaporation processes are active,


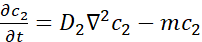
 (S3)

where *D*_2_ is the surface diffusivity of free carbon atoms on graphene, known to be much lower than the value of
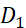
 for diffusion on the catalytic surface such as Cu (111).^15^ *m* is evaporation rate of free carbon atoms, assumed as the same value as that on substrate, and expected to play a minor role according to our additional simulation results with larger *m* values approaching 1. The second adiabatic BC is
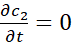
 at *x* = *L*.

The moving interface between these two regions, that is, the edge of GNRs, is modelled by tracking
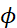
 using a level-set method in the Euler representation, *i.e.*


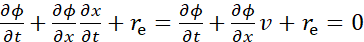
 (S4)


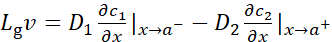
 (S5)

The velocity of interface migration *v* in Eq. S4 is determined by Eq. S5, which is reminiscent of the Stefan problem in the solid-liquid phase transition.^16-17^ However, the current problem is derived from mass conservation instead of that for energy. *L*_g_ is the difference in mass density upon phase changing, and *r*_e_ is the etching rate of carbon atoms removed from the as-grown GNRs.

***Results.*** A complete picture of the CVD graphene growth/etching processes on the liquid Cu substrate requires the solution of
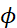
 from an evolutionary dynamics model (Eqs. S2-S5). The hydrogen flow rate controls *F* and *r*_e_.^18-19^ The *F* and *r*_e_ values were chosen according to the fact that the promotion of growth by elevating the chemical driving force is more significant than that of etching due to the enhanced decomposition of carbon sources in the etching-regulated regime (Figure 3a). However, at higher hydrogen flow rates, generation of free carbon *F* is suppressed in the decomposition reaction, while the etching rate *r*_e_ increases, corresponding to the etching-dominated regime (Figure 3a).

The modelling parameters and initial free carbon concentrations were chosen in reduced units as reported in our previous works.^20-21^ The graphene patterns regulated by the chemical environment (carbon sources, hydrogen flow rates) in experiments are also discussed in a wider range of parameters. In addition to *F* and *r*_e_, additional parameters include *D*_1_ = *D*_2_ = 10.0, *L* = 10.0, *m* = 1.0 × 10^−5^, *L*_g_ = 0.1. The initial carbon concentration is *c*_1_(*t* = 0) = 1.5, *c*_2_(*t* = 0) = 0.5, and 1.0 for the uncovered, covered substrate, and the interface, respectively.

Under the condition with the same concentration of carbon sources, the hydrogen flow rate controls the graphene growth/etching behaviours (Fig. S14). At a low hydrogen flow rate (*F* = 1.0 × 10^−2^, *r*_e_ = 5.0 × 10^−5^), the graphene edges extend to uncovered copper surfaces but could not reach coalescence as growth time increases, resulting in partially-etched graphene films. At an intermediate hydrogen flow rate (*F* = 5.0 × 10^−1^, *r*_e_ = 1.0 × 10^−4^), the neighbouring edges of graphene could merge over time, and fully-covered graphene films are predicted. At a very high hydrogen flow rate (*F* = 1.0, *r*_e_ = 1.0 × 10^−2^), etching dominates the whole process.


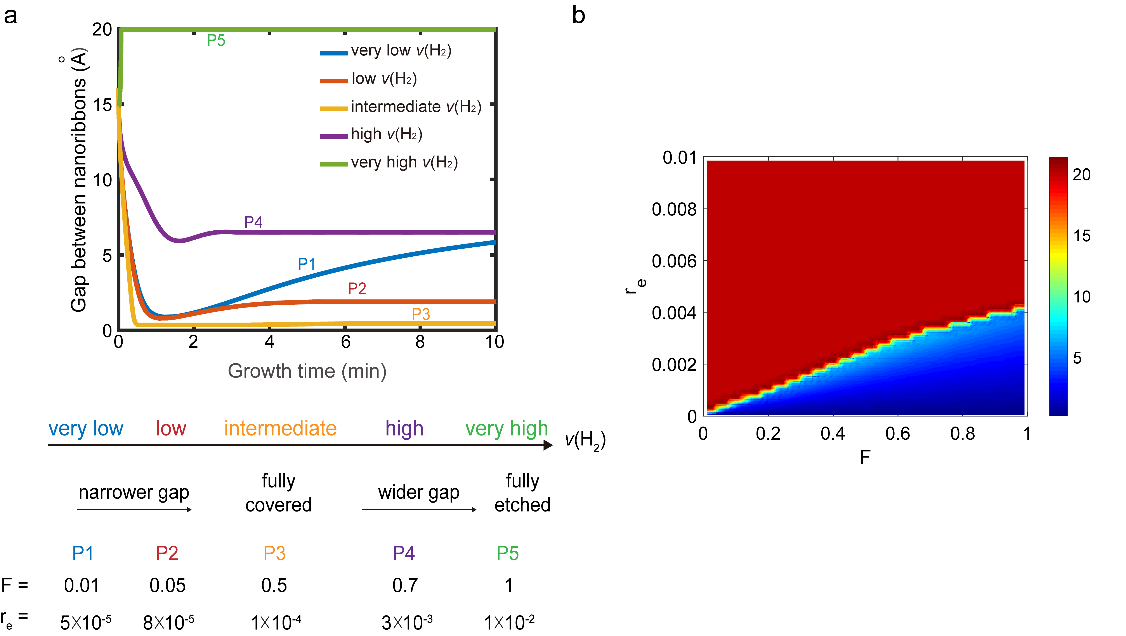


**Figure S14.** (a) Time evolution of graphene patterns at different hydrogen flow rates. Five representative parameter sets are chosen to illustrate the non-uniform dependence of gap distances between nanoribbons on the parameters *F* (the incoming rate of free carbon from deposition, adsorption and decomposition) and *r*_e_ (etching rate). (b) Modelling results of the gap distance (2*l*_s_) plotted as a function of *F* and *r*_e_ (Eqs. S2-S5).

***Discussion.*** Based on our theoretical model (Eqs. S2-S5) and the graphene patterns identified in experiments, we have additional arguments on the growth/etching processes. First, at the steady-state (
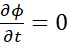
), the solution of Eq. S4 yields


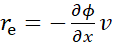
 (S6)

where
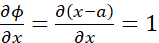
 by definition, and *r*_e_ is determined by the hydrogen flow rate. Consequently, at a certain hydrogen flow rate, the velocity of growth front is a constant in the steady state.

Second, mass conservation of carbon in the domain of solution requires that the generation rate of free carbon on the uncovered substrate with a span of 2*l*_s_ (Figure 4d) equals to the amount of carbon sources consumed by addition reactions at the growth front, that is,


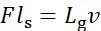
 (S7)

From **Eqs. S6** and **S7**, we see that the gap distance width (2*l*_s_) is determined by the values of *F* and *r*_e_ (Figure 4d). As a result, in the comb-like etching-regulated growth process, we predicted that 2*l*_s_ decreases with the hydrogen flow rate, in consistency with experimental observations.

**References**
